# Fracture of the bamboo spine (chronic ankylosing spondylitis) after cervical injury

**DOI:** 10.11604/pamj.2014.17.113.3888

**Published:** 2014-02-17

**Authors:** Ali Akhaddar, Mohcine Salami

**Affiliations:** 1 Department of Neurosurgery, Avicenne Military Hospital, Marrakech, Morocco; 2 Department of Neurosurgery, Mohammed V Military Teaching Hospital, Rabat, Morocco; 3 University of Mohammed V Souissi, Rabat, Morocco

**Keywords:** Bamboo spine, chronic ankylosing spondylitis, cervical injury

## Image in medicine

A 59-year-old man with a history of chronic ankylosing spondylitis for many years, developed neck pain and left cervico-brachial neuralgia following a road traffic accident sustained one week before. Plain radiographs of cervical spine were initially misinterpreted. On examination, he had severe neck pain on mobilization without any neurological deficits. Delayed cervical computed tomography scan showed ossification of the anterior longitudinal ligament, calcification of the intervertebral discs and complete vertebral fusion (so called bamboo spine) with transversal fracture at C5-C6 disc level (so called carrot-stick fracture) causing a luxation of the cervical spine with significant compromise in canal space (A and B). A transcranial spinal traction was performed followed by anterior decompression and stabilization via an anterolateral cervical approach. The outcome was favourable. Transverse fractures of the spine are rare in patients with ankylosing spondylitis and diagnosis should be considered following even minor trauma. These atypical unstable fractures occur because of the loss of flexibility and fragility of the osteoporotic spine. Early diagnosis for possible intervention is important because of the high mortality rate.

**Figure 1:**
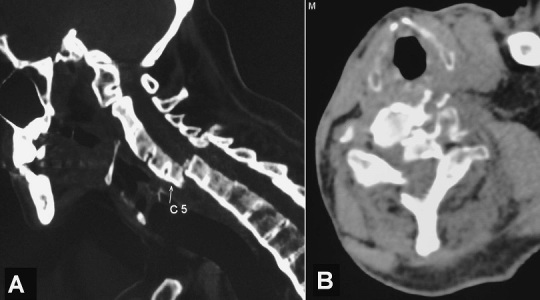
Cervical computed tomography scan on sagittal (A) and axial (B) views revealing ossification of the anterior longitudinal ligament, calcification of the intervertebral discs and complete vertebral fusion (bamboo spine) with transdiscal fracture at C5-C6 level causing a luxation of the cervical spine with significant compromise in canal space

